# Functional neurogenesis in the adult hippocampus: then and now

**DOI:** 10.3389/fnins.2014.00055

**Published:** 2014-03-27

**Authors:** Krishna C. Vadodaria, Sebastian Jessberger

**Affiliations:** ^1^Faculty of Medicine and Science, Brain Research Institute, University of ZurichZurich, Switzerland; ^2^Neuroscience Center Zurich, University of Zurich and ETH ZurichZurich, Switzerland

**Keywords:** pattern separation, emotion, cognition, dentate gyrus, hippocampal neurogenesis, subgranular zone

## Introduction

After two decades of research, the neurosciences have come a long way from accepting that neural stem/progenitor cells (NSPCs) generate new neurons in the adult mammalian hippocampus to unraveling the functional role of adult-born neurons in cognition and emotional control. The finding that new neurons are born and become integrated into a mature circuitry throughout life has challenged and subsequently reshaped our understanding of neural plasticity in the adult mammalian brain. It is now widely accepted that neurogenesis in the adult central nervous system occurs in multiple brain regions within the rodent brain, including the subventricular zone (SVZ) of the lateral ventricles and the subgranular zone (SGZ) of the dentate gyrus (DG). Since the discovery of ongoing neurogenesis in the adult brain, the field has been addressing questions regarding the cellular identity of adult NSPCs, the molecular pathways regulating maturation and integration of newborn neurons into preexisting circuitries, and how new neurons contribute to adult brain function. Technological advances over the last two decades such as targeted modulation (loss- and gain-of-function) of adult neurogenesis and refinements in behavioral testing paradigms have enabled us to begin addressing these questions directly. Here we give a brief overview of old and new studies examining the function of adult hippocampal neurogenesis (AHN) in the context of evolving technology, which has exponentially expanded our understanding of the neurogenic process in the adult mammalian brain.

## Early studies: from correlation to causality

Early on, the field went through a phase of correlating levels of AHN with performance in behavioral tests of hippocampus-dependent learning and memory, and affective behavior. Manipulations that increase AHN such as environmental enrichment, physical activity, and also treatment with certain antidepressants were found to enhance performance in spatial navigation tasks (e.g., Morris water maze; MWM) and in tests of anxiety-related behaviors (forced swim test, elevated plus maze) (Kim et al., 2012). Conversely, stress, aging, and inflammation, all of which negatively affect AHN, resulted in decreased performance in tasks of spatial navigation and emotion-related behaviors (Kim et al., 2012). Although correlative, these data generated in the late 1990s and early 2000s, suggested a role for AHN in hippocampus-dependent processes of cognition and emotion. The first studies attempting to show causal relationship between AHN and hippocampus-dependent behavior were published in the early 2000s, using the antimitotic drug methylazoxymethanol acetate (MAM) and focal irradiation of the hippocampus to ablate AHN. MAM-treated and focally irradiated mice showed impairments in hippocampus-dependent trace-conditioning and certain forms of long-term spatial memory (Shors et al., 2001; Snyder et al., 2005; Deng et al., 2009), suggesting that AHN was required for particular aspects of learning and memory. However, seemingly inconsistent findings from multiple studies with confounding variables such as incomplete elimination of neurogenesis and unwanted off-target effects (such as irradiation-induced inflammation) impeded a precise understanding of the contribution of AHN to hippocampal function (Deng et al., 2010).

## Functional hippocampal neurogenesis and evolving methodology

Significant advances in conditional gene targeting allowing the generation of transgenic mice and virus-based approaches enabled the selective targeting of adult hippocampal NSPCs and their neuronal progeny, and revealed not only the molecular pathways important for the different stages of neurogenesis, but also specific behavioral correlates of altered AHN (Saxe et al., 2006; Dupret et al., 2008; Jessberger et al., 2009; Deng et al., 2010; Ming and Song, 2011). Commonly used approaches include the expression of cell death-inducing genes (such as diphtheria toxin or its receptor and thymidine kinase that kills dividing cells upon gancyclovir injections), overexpression of pro-apoptotic genes (such as Bax), and expression of light-sensitive ion channels (such as channelrhodopsins enabling conditional depolarization or hyperpolarization of newborn neurons) in NSPCs and/or their neuronal progeny (Deng et al., 2010). Fewer methods have been utilized to genetically boost neurogenesis. One elegant approach has been to utilize transgenic mice where the pro-apoptotic gene BAX was conditionally deleted in nestin-expressing NSPCs (iBAX^nestin^), resulting in substantially enhanced levels of AHN (Sahay et al., 2011a). As compared to previous cytostatic drug- and irradiation-based strategies, these techniques improved temporal and tissue-specific control for ablating the desired neuronal population. Studies utilizing these strategies in combination with an array of behavioral tests have revealed novel roles for AHN. Together with correlational studies, genetic, and pharmacological approaches to manipulate levels of AHN are currently being used to understand the functional significance of AHN. Spatial discrimination tasks such as feared context, radial-arm maze, modified MWM, and the two-choice discrimination task have been utilized to test for a function of newborn neurons (Saxe et al., 2007; Clelland et al., 2009; Deng et al., 2009; Sahay et al., 2011a; Nakashiba et al., 2012) and there is now sufficient evidence suggesting that AHN plays a crucial role in the pattern separation functions of the DG (Treves et al., 2008; Yassa and Stark, 2011). The two-choice discrimination task where mice must discriminate between spatially proximate stimuli may become one of the behavioral tasks of choice (Clelland et al., 2009; Mctighe et al., 2009). Complementary approaches to knockdown AHN revealed selective deficits in this task and the radial arm maze. On the other hand, boosting AHN by genetically enhancing newborn neuron survival (using iBax^nestin^) enhances discrimination between similar contexts in a contextual fear-conditioning task (Sahay et al., 2011a). Notably, AHN becomes critical only when contexts/patterns become more similar and therefore more difficult to discriminate during recall; thus, AHN seems to be dispensable for discriminating between highly dissimilar contexts/patterns but crucial for computing and discerning highly similar input patterns. Transgenic strategies enabling selective ablation of young and adult-born DG neurons vs. mature DG granule neurons in combination with modifications of the MWM show that in the absence of mature neurons, separation between similar spatial contexts is enhanced, whereas, “completing” a pattern with only a subset of the cues is impaired (Nakashiba et al., 2012). These results highlight an interesting interplay between “newborn” and “old” neuronal populations, suggesting different yet complementary functions of pattern “separation” vs. “completion,” respectively. Collectively, studies from multiple labs provide evidence of a strong link between AHN and proposed pattern separation functions of the DG (Sahay et al., 2011b). Furthermore, recent data using novel transgenic mice and virus-based approaches (e.g., optogenetics and TK-based approaches) support the hypothesis that new neurons are particularly important for memory encoding and retrieval during a critical period 4–8 weeks after new neurons are born (Deng et al., 2009; Gu et al., 2012).

Recent reports also support a role for AHN in emotional control and affective behavior. These studies benefitted not only from novel methods to ablate AHN, but also refinements in testing paradigms for specific aspects of emotion-related behaviors (Samuels and Hen, 2011; Kheirbek et al., 2012). Particularly, irradiated and transgenic mice with diminished AHN exhibit signs of heightened stress response as observed in the food avoidance test (after acute stress), increased despair-like behavior in the forced swim test, and increased anhedonia in sucrose preference tests (Snyder et al., 2011). These deficits may be in part due to a dysfunctional regulation of the hypothalamic-pituitary-adrenal (HPA) axis that may lead to a disproportionate response to stress-inducing stimuli in mice with impaired AHN (Snyder et al., 2011). Interestingly, although ablation of AHN led to a heightened stress response along with behavioral correlates of depression-like behaviors, increasing neurogenesis by itself does not appear to be sufficient for promoting anxiolytic or antidepressant-like behaviors in the iBax mice (Sahay et al., 2011a). However, this may be due to a “ceiling” effect or due to limitations of current testing paradigms for examining “gain of function” in emotion-related behaviors.

## Functional AHN: open questions

Accumulating evidence over the last years has clearly demonstrated a role for AHN in hippocampus-dependent cognition and emotional control. However, it is currently unclear *how* exactly newborn neurons shape the DG circuitry and mediate DG-dependent pattern separation. A large number of open questions remain: how are individual patterns represented in the DG (Deng et al., 2013)? How does the hippocampal circuit “change” with the addition of each pattern-associated cohort of newborn neurons? How does top-down or cortical input regulate AHN and its function in learning new information? How much do newborn young neurons contribute to memory engrams in the DG? How do adult-born hippocampal neurons regulate the HPA axis, which contributes to the neurogenesis-associated regulation of anxiety-related behaviors? Do distinct subsets of newborn neurons contribute to pattern separation vs. emotional regulation role of the DG? Other questions pertain to the relevance of varying levels of AHN, basally, by environmental stimuli, and in the context of disease: How do variations in AHN contribute to individuality in exploratory behavior and could this be extended to humans (Freund et al., 2013)? How does aging regulate AHN and can boosting AHN alleviate age-related decline in aspects of cognition? Can AHN be harnessed for endogenous brain repair and restoration of neuronal function in diseases that is associated with diminished or altered AHN, such as major depression, epilepsy, Alzheimer's disease, and Parkinson's disease? Interestingly, recent findings that levels of hippocampal neurogenesis remain substantial even through the fifth decade of life in the adult human brain, opens up possibilities for doing functional studies in humans related to AHN (Spalding et al., 2013), for example by combining non-invasive imaging strategies together with DG-dependent behavioral paradigms (Brickman et al., 2011; Yassa and Stark, 2011; Dery et al., 2013). With the development of novel genetic tools there is great hope for answering these questions, however, it also seems plausible that we need to develop more refined and sensitive testing paradigms to closely examine AHN-dependent behaviors. In addition, it is clear that most genetic approaches are only suitable for studies using mice, limiting the possibility to use different species to broaden the relevance of the obtained findings. Thus, developing novel methods to measure and /or manipulate AHN in primates and even humans will be important to move the field toward biomedical relevance.

Be that as it may, the finding that the adult mammalian brain continuously generates new neurons throughout life has contributed significantly to our understanding of brain functioning and recent technological advances provide further impetus for studying the function of AHN in health and disease.
